# High-temperature thermoelectric properties of Na- and W-Doped Ca_3_Co_4_O_9_ system

**DOI:** 10.1039/c8ra01691g

**Published:** 2018-03-28

**Authors:** Uzma Hira, Li Han, Kion Norrman, Dennis Valbjørn Christensen, Nini Pryds, Falak Sher

**Affiliations:** Department of Chemistry and Chemical Engineering, SBA School of Science and Engineering, Lahore University of Management Sciences (LUMS) Lahore Pakistan fsher@lums.edu.pk +92 42 3560 8131; Department of Energy Conversion and Storage, Technical University of Denmark, Risø DTU Denmark nipr@dtu.dk +45 46 775752

## Abstract

The detailed crystal structures and high temperature thermoelectric properties of polycrystalline Ca_3−2*x*_Na_2*x*_Co_4−*x*_W_*x*_O_9_ (0 ≤ *x* ≤ 0.075) samples have been investigated. Powder X-ray diffraction data show that all samples are phase pure, with no detectable traces of impurity. The diffraction peaks shift to lower angle values with increase in doping (*x*), which is consistent with larger ionic radii of Na^+^ and W^6+^ ions. X-ray photoelectron spectroscopy data reveal that a mixture of Co^2+^, Co^3+^ and Co^4+^ valence states are present in all samples. It has been observed that electrical resistivity (*ρ*), Seebeck coefficient (*S*) and thermal conductivity (*κ*) are all improved with dual doping of Na and W in Ca_3_Co_4_O_9_ system. A maximum power factor (PF) of 2.71 × 10^−4^ W m^−1^ K^−2^ has been obtained for *x* = 0.025 sample at 1000 K. The corresponding thermoelectric figure of merit (*zT*) for *x* = 0.025 sample is calculated to be 0.21 at 1000 K, which is ∼2.3 times higher than *zT* value of the undoped sample. These results suggest that Na and W dual doping is a promising approach for improving thermoelectric properties of Ca_3_Co_4_O_9_ system.

## Abstract 2

The detailed crystal structures and high temperature thermoelectric properties of polycrystalline Ca_3−2*x*_Na_2*x*_Co_4−*x*_W_*x*_O_9_ (0 ≤ *x* ≤ 0.075) samples have been investigated.

## Graphical abstract

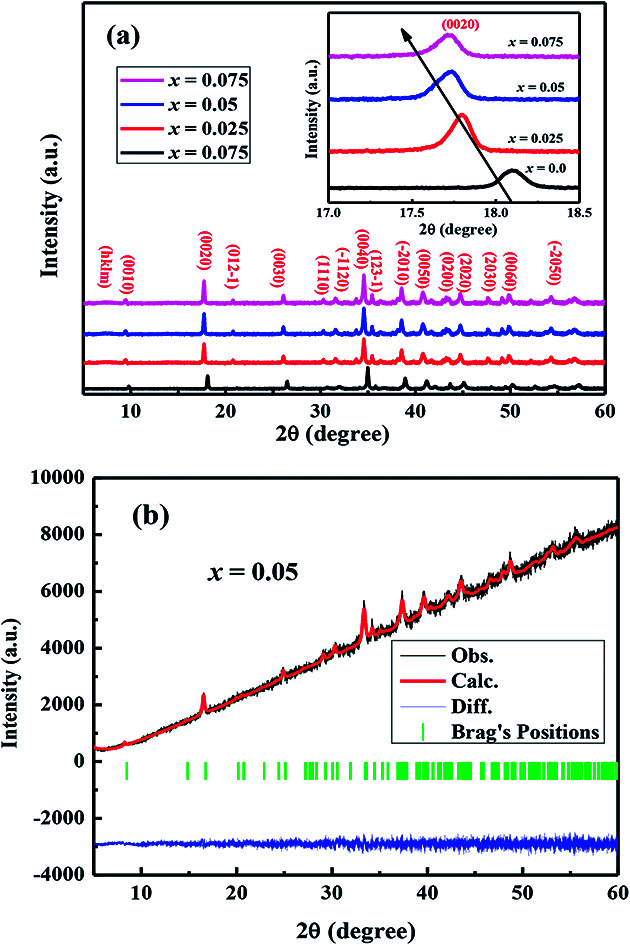

## 1. Introduction

Thermoelectric (TE) power generation from waste heat is considered as a promising renewable energy technology.^1,2^ TE devices convert thermal energy into electricity *via* the Seebeck effect, and electrical power into solid state refrigeration *via* the Peltier effect.^3^ In order to convert waste heat into electrical energy efficiently, good TE materials with high values of dimensionless figure of merit (*zT*) are required:^4^1
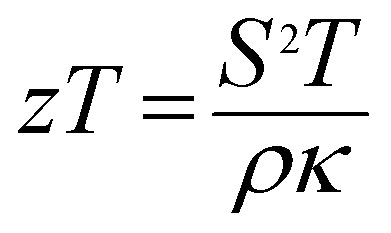
where *S* (V K^−1^) is the Seebeck coefficient, *T* (K) is the absolute temperature, *ρ* (Ωm) is the electrical resistivity, and *κ* (W m^−1^ K^−1^) is the thermal conductivity.

For practical high waste heat to electricity conversion efficiency devices, *zT* > 1 is an essential prerequisite. Therefore robust TE materials with large thermoelectric power factor: PF = *S*^2^/*ρ* and smaller thermal conductivity are required. In addition, TE materials must be stable in air at high operating temperatures over a long period of time and should be made of earth-abundant low cost elements. Conventional thermoelectric materials such as Bi_2_Te_3_ (*T*_max_ = 550 K), SiGe (*T*_max_ > 1300 K expensive and oxidation sensitive) and half-Heusler compounds (*T*_max_ = 850 K) do not meet all requirements for high temperature thermoelectric applications.^5–7^ Transition metal oxides are promising candidates and have been explored for their potential applications in high temperature thermoelectric devices. A number of transition metal oxides such as CaMnO_3_,^2^ Al-doped ZnO^8^ and Ta-doped SrTiO_3_ (ref. 9) show good thermoelectric properties and are stable in air at high temperatures of around 1000 K. Moreover, metal oxides can be synthesized from non-toxic and inexpensive precursors^10^ and can possibly segmented with non-oxide materials in TE modules to increase the efficiency of devices.^11^ Consequently, significant research efforts have been recently devoted to the development of thermoelectric generators (TEGs) for automotive applications.^12^ Among the transition metal oxides, misfit-layered cobaltites such as Na_*x*_CoO_2_,^13^ Ca_3_Co_4_O_9_,^14^ CuAlO_2_ (ref. 15) and Bi_2_Sr_2_Co_2_O_*x*_ (ref. 16) are considered to be promising p-type thermoelectric oxides for high temperature applications. Misfit-layered Ca_3_Co_4_O_9_ (abbreviated as C-349 in the following text) cobaltite is especially an interesting candidate material due to its good thermoelectric performance (*zT* ∼ 0.83 at 973 K for single crystal Ca_3_Co_4_O_9_ and ∼0.64 at 1073 K for heavily doped polycrystalline Ca_3_Co_4_O_9+*δ*_ materials with metallic nanoinclusions), and its high thermal and chemical stabilities in air.^17,18^

Ca_3_Co_4_O_9_ cobaltite has a monoclinic misfit structure with superspace group (*X*2/*m*(0*b*0)*s*0) crystal symmetry. C-349 compound is generally described as [Ca_2_CoO_3_][CoO_2_]_1.61_ and its high performance is linked with its unique layered crystal structure.^14^ It consists of two subsystems: a NaCl-type rocksalt (RS) Ca_2_CoO_3_ layer [subsystem 1] sandwiched between two CdI_2_-type (H) CoO_2_ hexagonal layers [subsystem 2].^19^ These two subsystems share the same *a*, *c* and *β* lattice parameters, and stack alternatively along the *c* axis. The mismatch of two unit cells results in dissimilar lattice parameters along the *b* axis *i.e.*, *b*_1_ [subsystem 1] and *b*_2_ [subsystem 2] with a ratio *b*_1_(RS)/*b*_2_(H) ∼ 1.61. The Ca_2_CoO_3_ (RS)-type block is an insulating layer whereas CoO_2_ (H) sheet is conductive.^20^

Recently, a number of research studies have focused on improving the TE performance of C-349 polycrystalline materials by using innovative synthesis methods such as spark plasma sintering (SPS),^21^ hot pressing,^22^ auto-combustion synthesis and sol–gel based electrospinning followed by SPS^23,24^*etc.* Chemical substitution of alternate metal cations at both Ca- and Co-sites of Ca_3_Co_4_O_9_ is another approach that has been used to fine tune the electrical and thermal transport properties of TE oxides. These studies include partial substitution of Na, Bi, Y, Ag, Nd, Sr and Pb ions^25–32^ at Ca-sites, which adjusts the carrier concentration without changing much the band structure of materials, and substitution of Fe, Mn, Cu, Ti, Ga, Mo, W and In ions^33–38^ at Co-sites with significant changes in the band structure and transport mechanism. In another research work, it was reported that doping of Na ions at Ca-sites resulted in decrease of electrical resistivity and as a consequence increase of thermoelectric power factor to ∼5.5 × 10^−4^ W m^−1^ K^−2^ at 1000 K, though thermal conductivity (*κ*) of these samples was still too high (4.0 W m^−1^ K^−1^), impeded the further improvement of *zT* values.^25^ On the other hand, high valence 4d and 5d transition metal-doped C-349 samples exhibited much smaller thermal conductivity with reasonably good *zT* values.^39^ There are some research studies on simultaneous substitution of two different metal cations at Ca- and Co-sites in C-349 system with significant improvement in TE properties with *zT* values of ∼0.20–0.25.^40–42^ However, there are no reports published on dual doping of Na and W metals in Ca_3_Co_4_O_9_ cobaltite as yet.

This prompted us to prepare a series of Ca_3−2*x*_Na_2*x*_Co_4−*x*_W_*x*_O_9_ (0 ≤ *x* ≤ 0.075) oxides by the conventional solid-state reaction method, and investigate their structural and high-temperature thermoelectric properties. We anticipated that Na and W dual doping in C-349 system would increase the Seebeck coefficient and electrical conductivity while thermal conductivity would decrease due to the W substitution. In this way, we expected to achieve much better *zT* values for these materials.

## 2. Experimental

Polycrystalline samples of Ca_3−2*x*_Na_2*x*_Co_4−*x*_W_*x*_O_9_ (0 ≤ *x* ≤ 0.075) series were prepared by the conventional solid-state reaction method. Stoichiometric quantities of CaCO_3_ (≥99.5%; Sigma-Aldrich), Co_3_O_4_ (≥99.5%; Sigma-Aldrich) and Na_2_WO_4_·2H_2_O (≥99.5%; Sigma-Aldrich) were ground, thoroughly mixed and pressed into pellets and initially sintered at 700 °C for 8 h. Sintered pellets were reground, pressed into pellets again and sintered twice at 900 °C for 8 h, with intermediate grinding and pelletizing, at a heating rate of 10 °C min^−1^ in air and slowly cooled down to room temperature.

Powder X-ray diffraction (XRD) data were collected in 2 theta range 5° ≤ 2*θ* ≤ 60° with a step size of 0.02° using a Bruker D8 Advanced diffractometer at room temperature with Cu K_α_ (*λ* = 1.5406 Å) radiation. Rietveld refinements of XRD data were performed using a computer program JANA2006.^43^ Surface morphology of samples was studied using a FEI Nova NanoSEM 450 scanning electron microscope (SEM). X-ray photoelectron spectroscopy (XPS, Thermo Electron Limited, Winsford, UK) was used to examine the oxidation states of Co and W ions in C-349 based materials. Analyses were performed using a monochromatic (Al-Kα) X-ray source at room temperature with a take-off angle of 90° from the surface plane. High-resolution Co 2p and W 4f XPS spectra were recorded using 50 eV detector pass energy and 10 scans. The binding energies were assessed by referencing to the Au 4f peak at 84.0 eV. Hall measurements were carried out at room temperature by using van der Pauw method with a (5.08 T) superconducting magnet.

The Seebeck coefficient (*S*) and electrical resistivity (*ρ*) were simultaneously measured from room temperature to 1000 K with an ULVAC-RIKO ZEM3 under a low pressure helium atmosphere. The thermal diffusivity (*α*) was measured with (NETZSCH LFA-457) laser flash system under vacuum. The heat capacity (*C*_p_) was estimated using temperature independent Dulong–Petit law. The thermal conductivity (*κ*) was calculated using equation (*κ* = *α*·*ρ*·*C*_p_), where *C*_p_, *ρ* and *α* are the specific heat capacity, mass density and thermal diffusivity, respectively. Mass density of the samples was measured by Archimedes method using water with a few drops of surfactant.

## 3. Result and discussion

### 3.1. Crystal structure and surface morphology

**Fig. 1:**
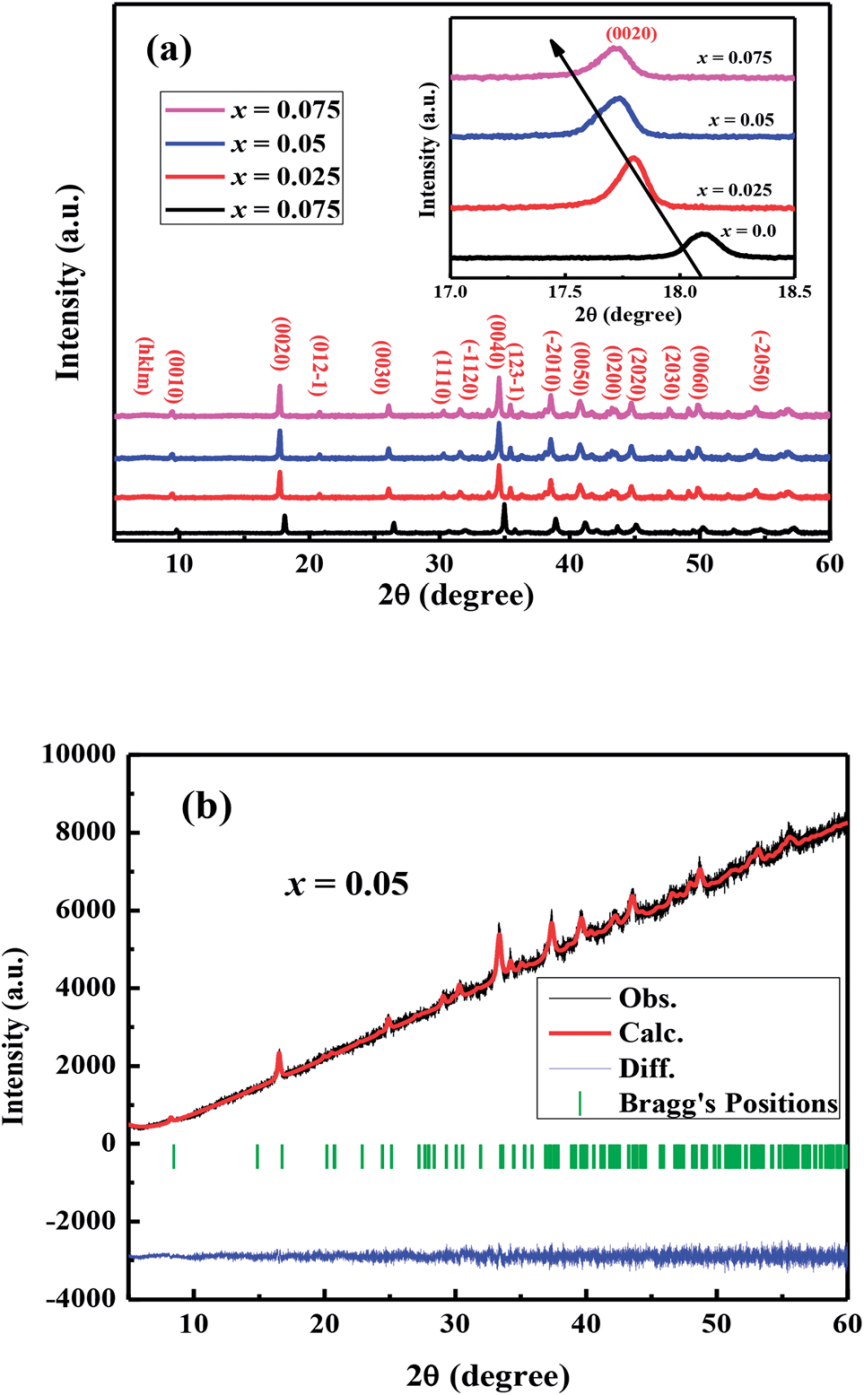
(a) XRD patterns of Ca_3−2*x*_Na_2*x*_Co_4−*x*_W_*x*_O_9_ (0 ≤ *x* ≤ 0.075) samples; inset: the shift of (0020) peaks with change of doping content (*x*). (b) Rietveld refined XRD pattern of Ca_2.9_Na_0.1_Co_3.95_W_0.05_O_9_ sample.

**Fig. 2:**
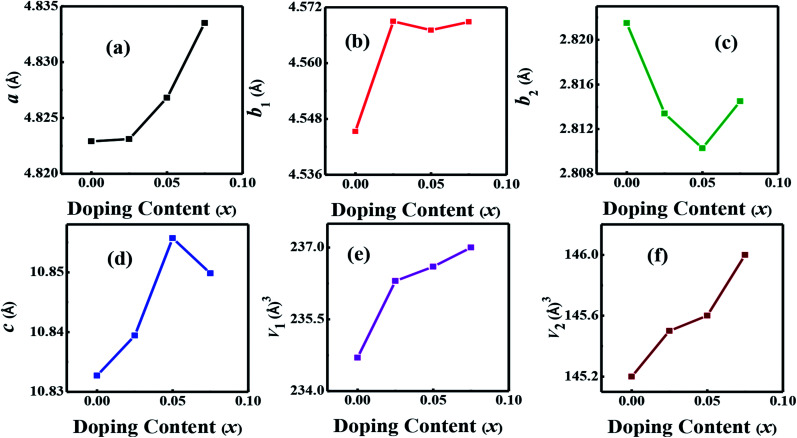
Structural parameters *a*, *b*_1_, *b*_2_, *c*, *V*_1_ and *V*_2_ as a function of doping content (*x*) for Ca_3−2*x*_Na_2*x*_Co_4−*x*_W_*x*_O_9_ (0 ≤ *x* ≤ 0.075) series.

The crystal structures of Ca_3−2*x*_Na_2*x*_Co_4−*x*_W_*x*_O_9_ (0 ≤ *x* ≤ 0.075) samples were analyzed by collecting the powder X-ray diffraction data at room temperature. The diffraction peaks in XRD patterns of all samples (Fig. 1(a)) are identical to the standard JCPDS card (21-139) of C-349 system,^44^ indicating the formation of phase pure compounds. The enlarged portions of (0020) diffraction peaks are presented in inset of Fig. 1(a) to illustrate the effect of Na and W dual doping on C-349 crystal structure. It can be clearly seen that the diffraction peaks shift to lower 2*θ* values with increase in doping content (*x*). The XRD data was Rietveld refined using a computer program JANA 2006 (ref. 43) in the superspace group *X*2/*m*(0*b*0)*s*0 and the resulting structural parameters are listed in Table 1. The refined XRD pattern of *x* = 0.05 sample is shown in Fig. 1(b), as an example. It can be seen from Table 1 and Fig. 2 that the lattice parameters *a*, *b*_1_, *c* and unit cell volumes (*V*_1_ and *V*_2_) all slightly increase with increase in doping content (*x*), which is consistent with the observed shifting of diffraction peaks to lower 2*θ* values. However, the values of *b*_2_ decrease with increase in doping suggesting that there is an increase of crystallographic distortion. This trend in the lattice parameters with doping is in agreement with the observation that ionic radii of Na^+^ (1.02 Å) and W^6+^ (0.6 Å) ions are larger than the corresponding Ca^2+^ (1.0 Å) and Co^3+^ (0.545 Å)/Co^4+^ (0.53 Å) ions in the six coordination number,^45^ respectively. The quality factors like GOF, *R*_wp_ and *R*_p_ (Table 1) are all within the acceptable statistical range, suggesting that the Rietveld refinements of samples are reliable.

**Table 1:** Crystallographic parameters for Ca_3−2*x*_Na_2*x*_Co_4−*x*_W_*x*_O_9_ (0 ≤ *x* ≤ 0.075) samples obtained from the Rietveld refinement analysis of powder X-ray diffraction data^a^

Composition Ca_3−2*x*_Na_2*x*_Co_4−*x*_W_*x*_O_9_	*x* = 0.0	*x* = 0.025	*x* = 0.05	*x* = 0.075
**Lattice parameters**				
*a* (Å)	4.8229(2)	4.8231(5)	4.8268(5)	4.8335(2)
*b*_1_ (Å)	4.5453(2)	4.5690(8)	4.5671(9)	4.5689(8)
*b*_2_ (Å)	2.8215(3)	2.8134(3)	2.8103(8)	2.8145(3)
*c* (Å)	10.8215(9)	10.8394(2)	10.8557(2)	10.8498(2)
*β* (deg)	98.807(3)	98.329(1)	98.582(8)	98.396(4)
*δ* (*b*_1_/*b*_2_)	1.61	1.62	1.62	1.62
*V*_1_ (Å)^3^	234.7(4)	236.3(4)	236.6(4)	237.0(4)
*V*_2_ (Å)^3^	145.2(7)	145.5(7)	145.6(7)	146.0(7)

**Reliability factors**				
*R*_wp_ (%)	3.99	2.94	2.41	1.97
*R*_p_ (%)	3.25	2.34	1.93	1.51
GOF	3.29	2.44	1.84	1.97

a *b*_1_ and *b*_2_ are the *b*-axis lattice parameter for [Ca_2_CoO_3_] and [CoO_2_] subsystems, respectively.

**Fig. 3:**
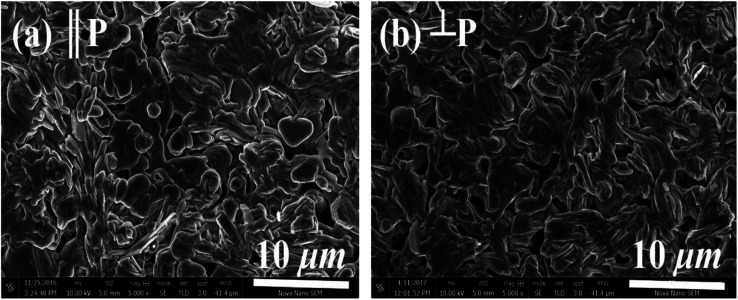
SEM micrographs of (a) parallel (‖P) and (b) perpendicular (⊥P) surfaces for *x* = 0.05 sample.

The morphology of samples in two different directions, parallel (‖p) and perpendicular (⊥p), to the pellets pressure axis (Fig. 3) were studied by the scanning electron microscopy in order to find out if there is any micro-dimensional anisotropy in these layered materials. The grain morphology in both parallel (‖p) and perpendicular (⊥p) directions of the pressure axis seems to be almost identical, suggesting that there is no or negligible anisotropy that can be observed on a micrometer scale. The SEM images show the plate-like crystal grains morphology, which is a typical feature of materials, including C-349 system, that are prepared by the conventional solid state chemistry method.^46^ Close inspection of the SEM micrograph for *x* = 0.025 sample reveals that crystal grains are larger (∼2.52 μm) and more compact than crystal grains of other samples (0.83–1.45 μm). The measured densities for all samples are in the range ∼86–94% of the theoretical density (Table 2).

**Table 2:** Average grain size, bulk density, carrier mobility (*μ*_300 K_), activation energy (*E*_a_) and important thermoelectric parameters are listed

Composition Ca_3−2*x*_Na_2*x*_Co_4−*x*_W_*x*_O_9_	*x* = 0.0	*x* = 0.025	*x* = 0.05	*x* = 0.075
Average grain size (μm)	0.83 (±0.002)	2.52 (±0.09)	1.45 (±0.005)	0.80 (±0.007)
Density (g cm^−3^)	3.801 (±0.02)	4.09 (0.045)	3.98 (±0.032)	3.844 (±0.068)
Theoretical density (g cm^−3^)	4.37	4.37	4.38	4.39
Relative density (%)	86.27	94.67	90.87	87.56
Mobility, *μ*_300 K_ (cm^2^ V^−1^ s^−1^)	0.64	0.82	0.74	0.65
*ρ*_*T* = 1000 K_ × 10^−5^ (Ωm)	23.98	13.07	16.09	27.54
*E*_a_ (meV)	49	98	103	76
*S*_*T* = 1000 K_ (μV K^−1^)	174.99	182.72	192.67	215.99
*κ*_*T* = 1000 K_(W m^−1^ K^−1^)	1.367	1.266	1.323	1.343
*zT*_*T* = 1000 K_	0.093	0.21	0.18	0.13

**Fig. 4:**
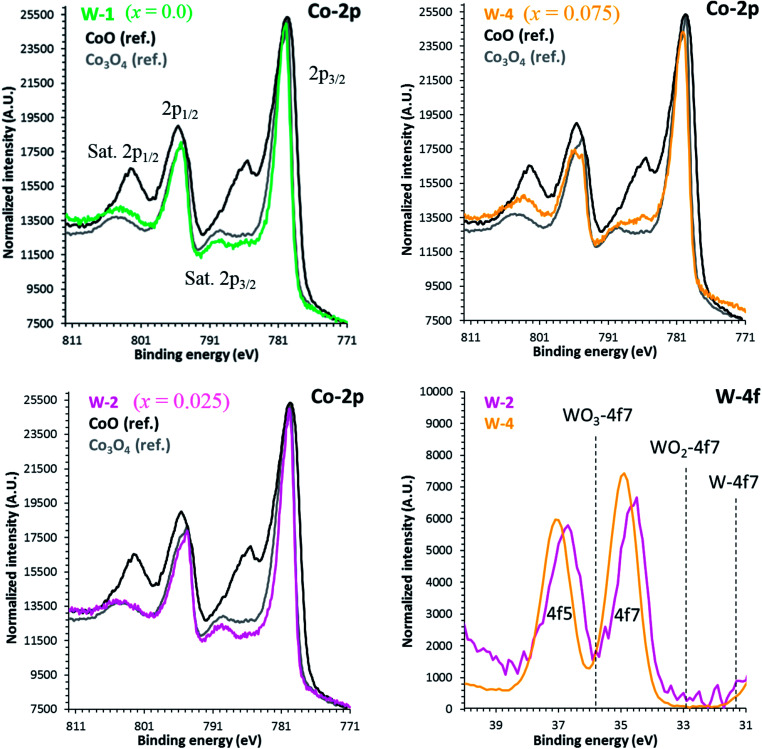
High resolution XPS spectra of Co 2p and W 4f for C-349 doped samples. Reference positions are taken from the NIST database.

The binding energies of Co 2p and W 4f sub-shells of selected samples were estimated from the high resolution XPS measurements as shown in Fig. 4. As reported elsewhere, the XPS spectrum of Co 2p splits into two parts, Co 2p_3/2_ and 2p_1/2_ with an intensity ratio of approximately (2 : 1) due to the spin–orbit coupling.^47^ The line shapes of both Co 2p_3/2_ and 2p_1/2_ spectra are similar to the reported results in literature.^48^ The main peaks corresponding to the Co 2p_3/2_ energy are located at 778.8 eV, 779.69 eV and 781.0 eV corresponding to *x* = 0.0, 0.025 and 0.075 samples, respectively. Shake-up satellite peaks due to the metal-to-ligand charge transfer processes at higher binding energies than the 2p_3/2_ and 2p_1/2_ main peaks are also detected. The observed variations in Co 2p_3/2_ binding energies can be explained by the larger electronegativity of tungsten (2.36, Pauli scale) than cobalt (1.88, Pauli scale).^49^ With careful analysis of XPS data, we can predict that Co ions have three types of valence states: Co^2+^, Co^3+^ and Co^4+^ in all samples. However, the average valence state of Co is most likely between 3+ and 4+ as reported elsewhere.^50,51^ The observed increase in binding energies of Co 2p_3/2_ peaks suggests that the relative population of Co^3+^ ions is decreasing with increase in doping. The 4f_5_ and 4f_7_ peaks for W ions closely resemble with the reference peaks of WO_3_, indicating that they are present in W^6+^ valence states in all samples.

### 3.2. Thermoelectric properties

**Fig. 5:**
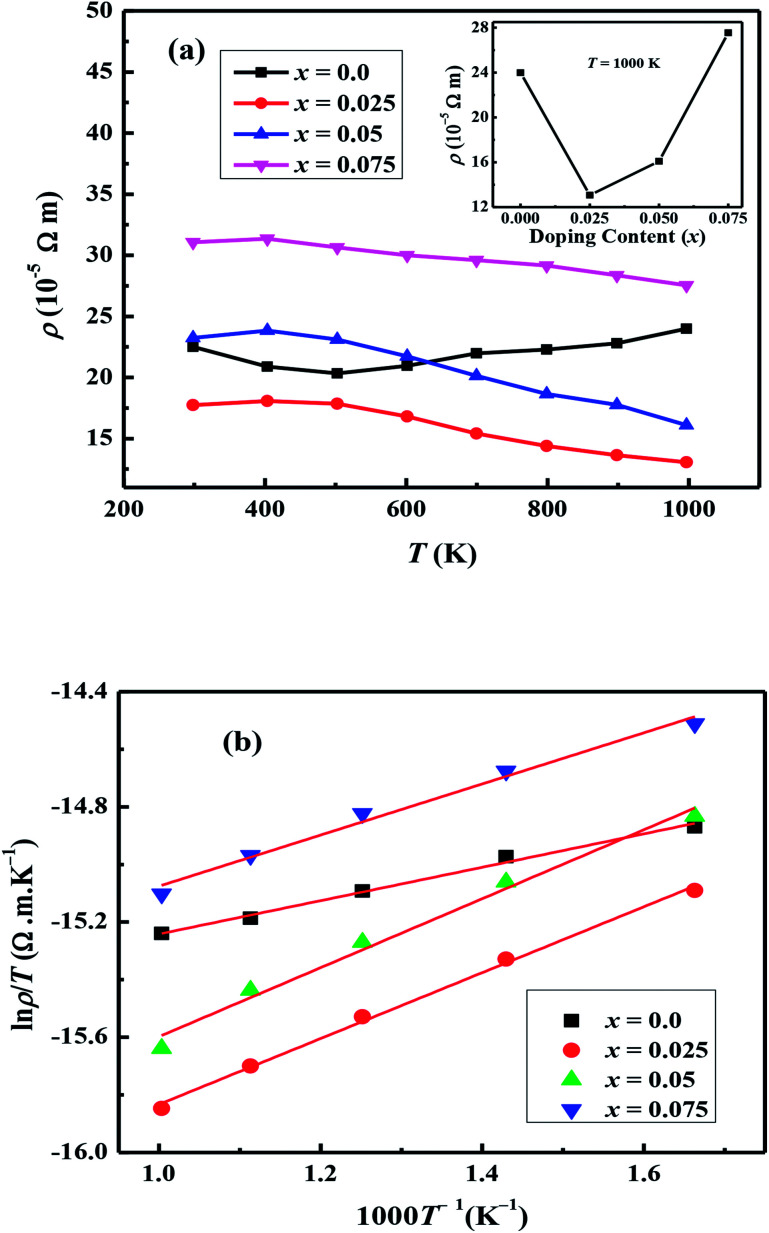
(a) Electrical resistivity (*ρ*) for Ca_3−2*x*_Na_2*x*_Co_4−*x*_W_*x*_O_9_ (0 ≤ *x* ≤ 0.075) samples as a function of temperature; inset shows doping content (*x*) dependence of *ρ* at 1000 K. (b) Linear fits of ln(*ρ*/*T*) *vs.* 1000/*T* for Ca_3−2*x*_Na_2*x*_Co_4−*x*_W_*x*_O_9_ (0 ≤ *x* ≤ 0.075) samples.

The temperature dependent electrical resistivities, *ρ*(*T*), as a function of Na and W co-doping (*x*) are shown in Fig. 5(a). The *ρ*(*T*) curve for *x* = 0.0 sample exhibits a semiconducting like behavior (d*ρ*/d*T* < 0) from room temperature to around 500 K, and then shows a transition to metallic like behavior (d*ρ*/d*T* > 0) from 600 K onwards. This kind of behavior in resistivity of C-349 system has been previously attributed to the spin-state transition,^52^ removal of oxygen atoms from porous layered cobaltites^38^ and structural distortion in Ca_2_CoO_3_ sheets.^53^ On the other hand, all doped samples show metallic behavior at low temperatures before showing a transition to semiconducting behavior above 400 K. The absolute values of resistivity at 1000 K for *x* = 0.025 and 0.05 samples are smaller than that of undoped sample, but higher for *x* = 0.075. This shows that dual doping of small amounts of Na and W has beneficial effect on resistivity of our samples.

We can describe the high temperature electrical resistivity of cobaltites using the small polaron hopping model,^54^ which is given by the following relation:2
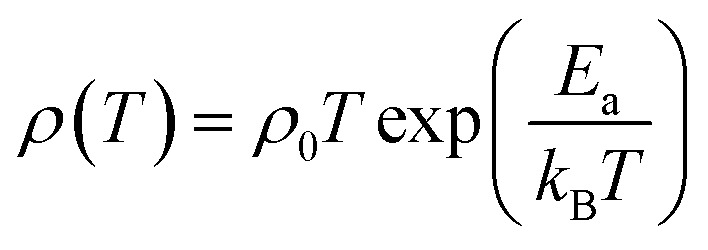
where *ρ*_0_ is a constant factor called the residual resistivity, *k*_B_ is the Boltzmann constant and *E*_a_ is the activation energy of electrical conductivity for polaron hopping.

The linear fits of ln(*ρ*/*T*) *versus* 1/*T* above 600 K, as shown in Fig. 5(b), suggest that the small polaron hopping model applies well to the electrical resistivity of these materials. The slopes of straight lines (*E*_a_/*k*_B_) were used to estimate the activation energies for all samples as listed in Table 2. It has been observed that *E*_a_ values for doped samples are relatively higher than the pristine C-349 system. This suggests that energy demand for carriers to jump from the top of valence band to the bottom of conduction band, in general, increases with doping in our samples. However, this variation could also be due to the creation of some in-gap states which would change with doping. As discussed elsewhere, hoping of carriers occurs between Co^3+^ and Co^4+^ in CoO_2_ layer and as a consequent the ratio of Co^3+^ and Co^4+^ ions directly affects the hopping distance in these materials.^55^ We anticipate that concentration of Co^4+^ ions would decrease with increase in doping of W^6+^ ions resulting in increase of hopping distance and therefore increase in activation energies with doping. Similar results have been reported for the activation energies of Fe, Ag, Gd and Y doped misfit layered cobaltites.^41,55,56^

**Fig. 6:**
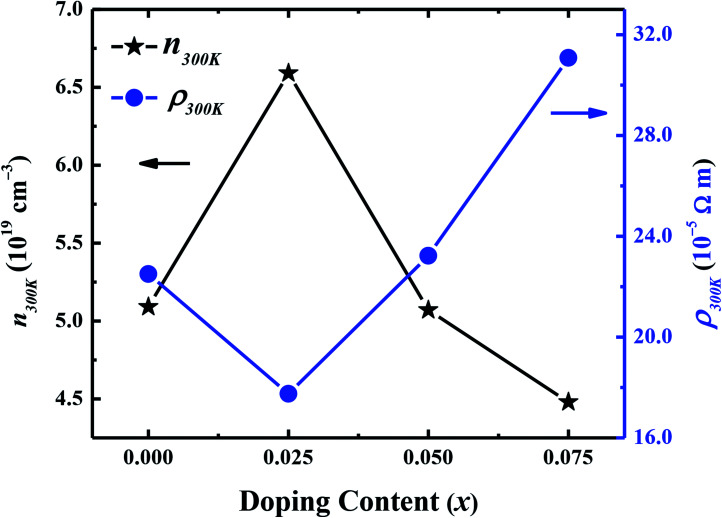
Room temperature carrier concentration (*n*_300 K_) and electrical resistivity (*ρ*_300 K_) as a function of doping (*x*) for Ca_3−2*x*_Na_2*x*_Co_4−*x*_W_*x*_O_9_ (0 ≤ *x* ≤ 0.075) samples.

Hall effect measurements were carried out at room temperature for all samples, to determine the carrier concentration and their mobility as a function of dopant. It has been observed that carrier concentration (*n*_300 K_) initially increases from 5.12 × 10^19^ cm^−3^ (*x* = 0.0) to 6.59 × 10^19^ cm^−3^ (*x* = 0.025) with doping and then decreases again to 4.48 × 10^19^ cm^−3^ (*x* = 0.075) with further increase in doping content as shown in Fig. 6. This increase in carrier concentration at low doping level is probably due to the substitution of Na^+^ for Ca^2+^ ions in C-349 system which results in increase of the number of hole carriers. With further increase in doping content (*x*), structural distortions and electron-doping like behavior of W^6+^ ions, due to the higher valence state of W^6+^ than Co^3+^ and Co^4+^ ions, start to dominate and result in decrease of carrier concentration. The carrier mobilities (*μ*_300 K_) also follow the same trend and decrease to a value of 0.65 cm^2^ V^−1^ s^−1^ with increase in doping after showing the maximum value for *x* = 0.025 sample (Table 2). These trends in *n*_300 K_ and *μ*_300 K_ together with the larger grain sizes (Table 2) of Na and W dual doped samples can be used to explain the electrical resistivity of these materials. With the largest values of *n*_300 K_, *μ*_300 K_ and grain sizes, the *x* = 0.025 sample has the lowest value of electrical resistivity and then *ρ* increases with increase in doping according to the equation: 1/*ρ* = *neμ*.

**Fig. 7:**
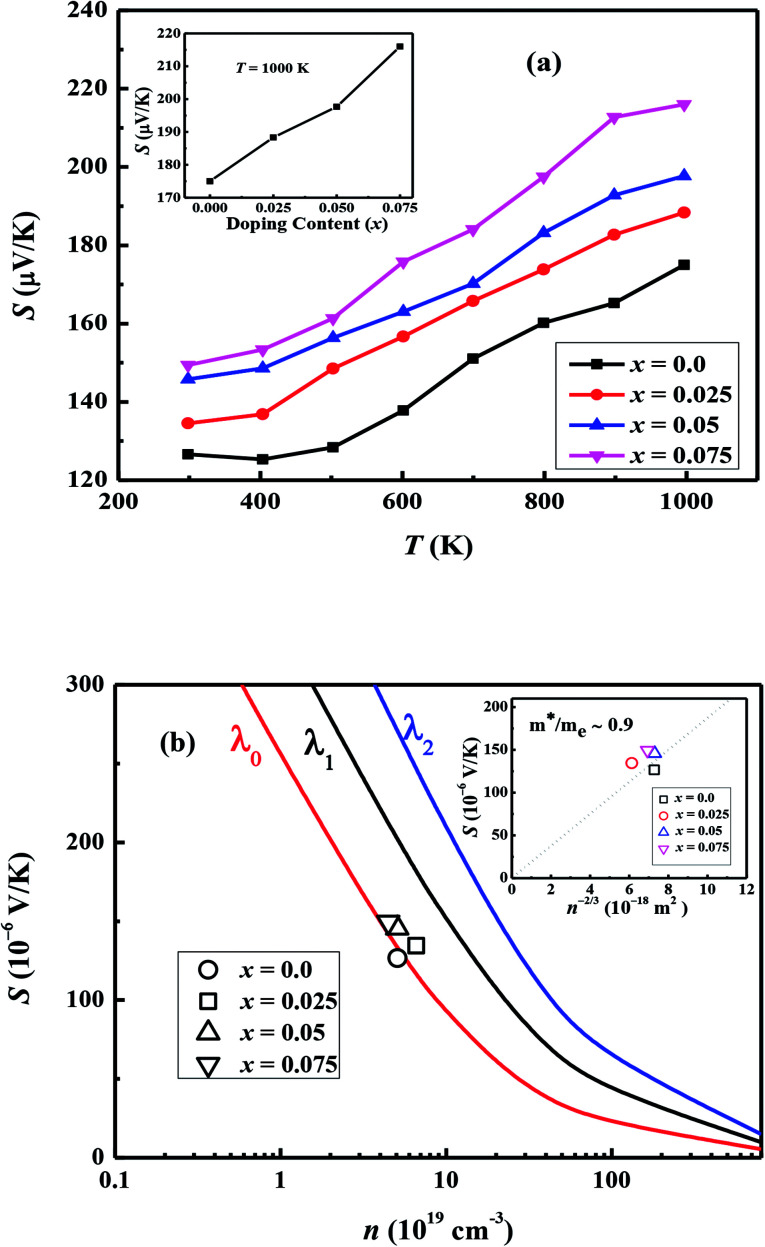
(a) Thermopower (*S*) for Ca_3−2*x*_Na_2*x*_Co_4−*x*_W_*x*_O_9_ (0 ≤ *x* ≤ 0.075) samples as a function of temperature; inset shows doping content (*x*) dependence of *S* at 1000 K. (b) Thermopower (*S*) plotted as a function of carrier concentration (*n*); solid colored lines are the calculated values for (*λ* = 0, 1 & 2) representing electron scattering by acoustic phonons, optical phonons and ionized impurities respectively. Inset: plot of *S vs.* (*n*^−2/3^); diagonal line corresponding to the Pisarenko relation with m*/*m*_e_ ∼ 0.9.

Fig. 7(a) shows the temperature dependence of thermopower (*S*) of the Ca_3−2*x*_Na_2*x*_Co_4−*x*_W_*x*_O_9_ samples. The sign of *S* is positive for all samples suggesting that holes are the majority charge carriers. The values of *S* increase with increase in temperature for all samples. It is also evident from Fig. 7(a) (inset) that thermopower values increase with increase in doping content (*x*). The maximum value of 216 μV K^−1^ at 1000 K for *x* = 0.075 sample is higher than previously reported *S* values for Na doped C-349 system (187 μV K^−1^) at this temperature.^25,40^ As discussed above, *x* = 0.025 sample shows the largest values of *n*_300 K_ and *μ*_300 K_, and then values of these quantities decrease with further increase in doping. This is consistent with the observed behavior of *S* with doping in our samples.^26,28^ The contribution of carrier concentration and carrier mobility, *μ*(*ε*) in describing *S* is given by the Mott's formula (originated from the Sommerfeld expansion).^57^3
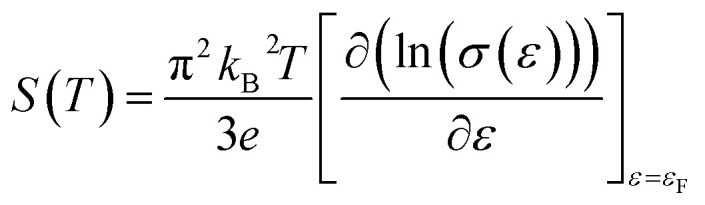


By using [*σ* = *enμ*(*ε*)] in eqn (3), we can get:4
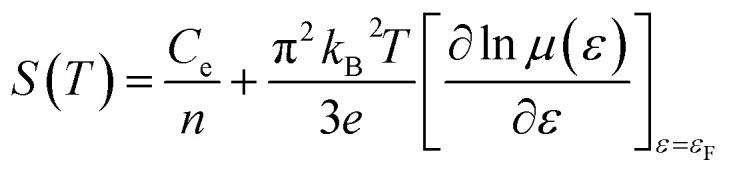
where *C*_e_ = (π^2^*k*_B_^2^*T*/3*e*)*Ψ*(*ε*) and *n*, *μ*(*ε*) *C*_e_ and *Ψ*(*ε*) are the carrier concentration, energy correlated carrier mobility, electronic specific heat and density of state (DOS), respectively. There are three possible reasons associated with this increase in *S* values: (1) the Drude model predicts that first term 
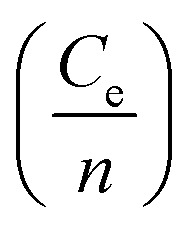
 in above equation is dominant.^58^ In Na and W dual doped samples, carrier concentration (*n*) decreases with increase in doping (*x*) and therefore thermopower (*S*) increases. However, *x* = 0.0 sample does not follow this trend suggesting that the electronic specific heat (*C*_e_) and second term in above equation are the dominated factors here. Wang *et al.*, reported that Fe doping in C-349 system increases the carrier concentration (*n*) and the electronic specific heat (*C*_e_), but the effect of *C*_e_ dominates over *n* which results in larger *S* values for doped samples.^59^ (2) We could assume that slope of the density of states at Fermi level is the main contribution to the second part of the above equation for undoped sample. (3) Partial substitution of W^6+^ for Co^3+^/Co^4+^ ions decreases hole carriers and thus results in an increase of thermopower.

According to the Pisarenko relation for degenerated semiconductors:5
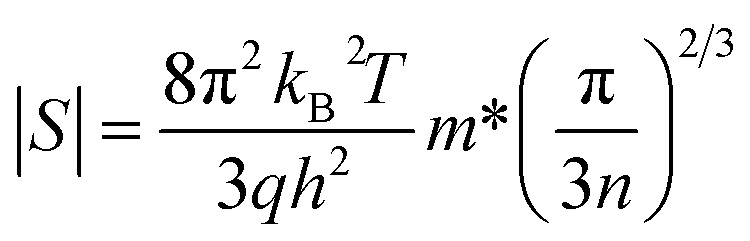
where *k*_B_, *h*, *q* and m* are the Boltzmann constant, Plank's constant, unit charge of electron and effective mass of carriers respectively. We have calculated a value of m*/*m*_e_ ∼ 0.9 for all samples by plotting room temperature *S* values *vs. n*^−2/3^ as shown in inset of Fig. 5(b). We can apply a simple parabolic band model by using the measured *n* and estimated m* values as described by the following equations:^60^6
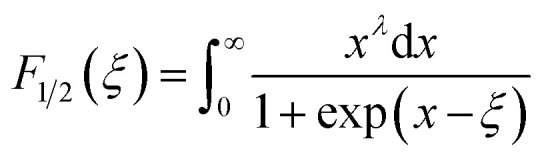
7
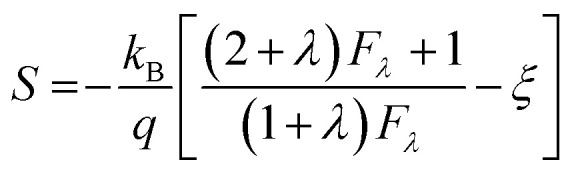
8
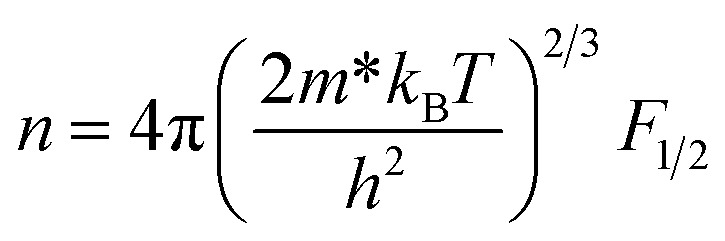
where *F*_1/2_(*ξ*) is the Fermi integral, *ξ* is the reduced electrochemical potential, *λ* is a scattering parameter and its value is taken 0 for acoustic phonon scattering, 1 for optical phonons scattering, and 2 for ionized impurity scattering.^60^ The calculated *S* values at room temperature as a function of carrier concentration (*n*) are shown in Fig. 7(b). Three different scattering mechanisms are represented by three straight lines in this plot. The measured and calculated values of *S* match very well when we take *λ* = 0, which suggests that the acoustic phonon scattering is the dominant scattering mechanism for all samples.

**Fig. 8:**
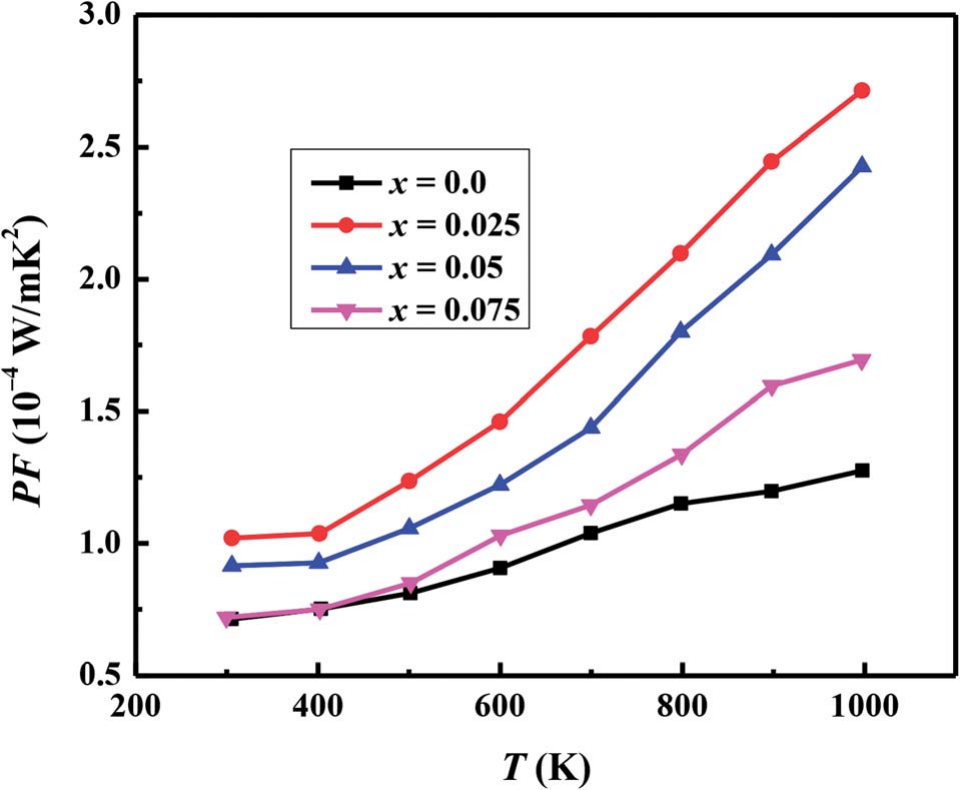
Power factor (PF) for Ca_3−2*x*_Na_2*x*_Co_4−*x*_W_*x*_O_9_ (0 ≤ *x* ≤ 0.075) samples as a function of temperature.

We have used the electrical resistivity and thermopower values to calculate the thermoelectric power factor PF = *S*^2^/*ρ* for all samples as shown in Fig. 8. The PF values increase with increase in temperature for all samples due to increase of thermopower with temperature. We can also see from Fig. 8 that PF values are significantly improved with Na and W dual doping in C-349 system. Among all doped samples, *x* = 0.025 sample exhibits the highest PF of 2.71 × 10^−4^ W m^−1^ K^−2^ at 1000 K which is about 2.3 times more than PF, 1.27 × 10^−4^ W m^−1^ K^−2^, of undoped sample. The PF obtained in this work is higher than the previously reported value of ∼2.1 × 10^−4^ W m^−1^ K^−2^ at 1073 K for Ca_3_Co_3.97_Cu_0.03_O_9_ sample prepared by the conventional solid state reaction method.^61^

#### 3.2.1. Thermal conductivity

**Fig. 9:**
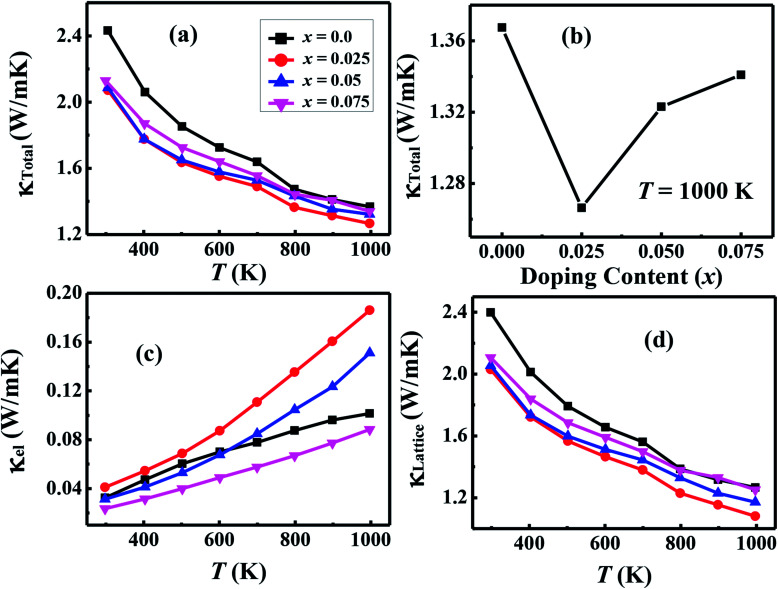
(a) Total thermal conductivity (*κ*_Total_ = *κ*_el_ + *κ*_Lattice_); (b) doping content (*x*) dependence of *κ*_Total_ at 1000 K; (c) electronic part of thermal conductivity (*κ*_el_) and (d) lattice part of thermal conductivity (*κ*_Lattice_) for Ca_3−2*x*_Na_2*x*_Co_4−*x*_W_*x*_O_9_ (0 ≤ *x* ≤ 0.075) samples as a function of temperature.

The temperature dependent total thermal conductivity (*κ*_Total_) for Ca_3−2*x*_Na_2*x*_Co_4−*x*_W_*x*_O_9_ samples is shown in Fig. 9(a). We can clearly see that *κ*(*T*) decreases with increase in temperature for all samples in the measured temperature range. For *x* = 0.0 sample, measured value of *κ* at 1000 K is 1.36 W m^−1^ K^−1^ and it decreases to 1.26 W m^−1^ K^−1^ for *x* = 0.025 sample. On further increase in doping, *κ*_1000 K_ slightly increases again but its value remains lower than the pristine C-349 system. In order to understand the observed changes, we have investigated the contribution of electronic (*κ*_el_) and lattice (*κ*_Lattice_) parts of the thermal conductivity, separately. Fig. 9(b) shows the values of *κ*_el_ as determined from the experimentally measured *ρ* values by using the Wiedemann–Franz law (*κ*_el_ = *LT*/*ρ*), where *L* is the Lorentz number and its value is 2.44 × 10^−8^ W Ω K^−2^ for free electrons.^3^ The values of *κ*_Lattice_ were calculated using the relation *κ*_Lattice_ = *κ*_Total_ − *κ*_el_ and are shown in Fig. 9(c). It is evident from plot that *κ*_Lattice_, and not *κ*_el_, is the major contributing factor to total thermal conductivity of our samples. Hence, changes in *κ*_Total_ with increase in doping content (*x*) are mainly originated from the changes in *κ*_Lattice_.^33^ We can attribute these changes in *κ*_Lattice_ to the larger ionic radius of W^6+^ than Co^3+^/Co^4+^ ions resulting in structural distortions, and therefore increase in phonon scattering. However, we cannot rule out some other unexplained microstructural aspects of these materials that can also be responsible for irregular behavior in thermal conductivity of doped samples.

#### 3.2.2. Figure of merit

**Fig. 10:**
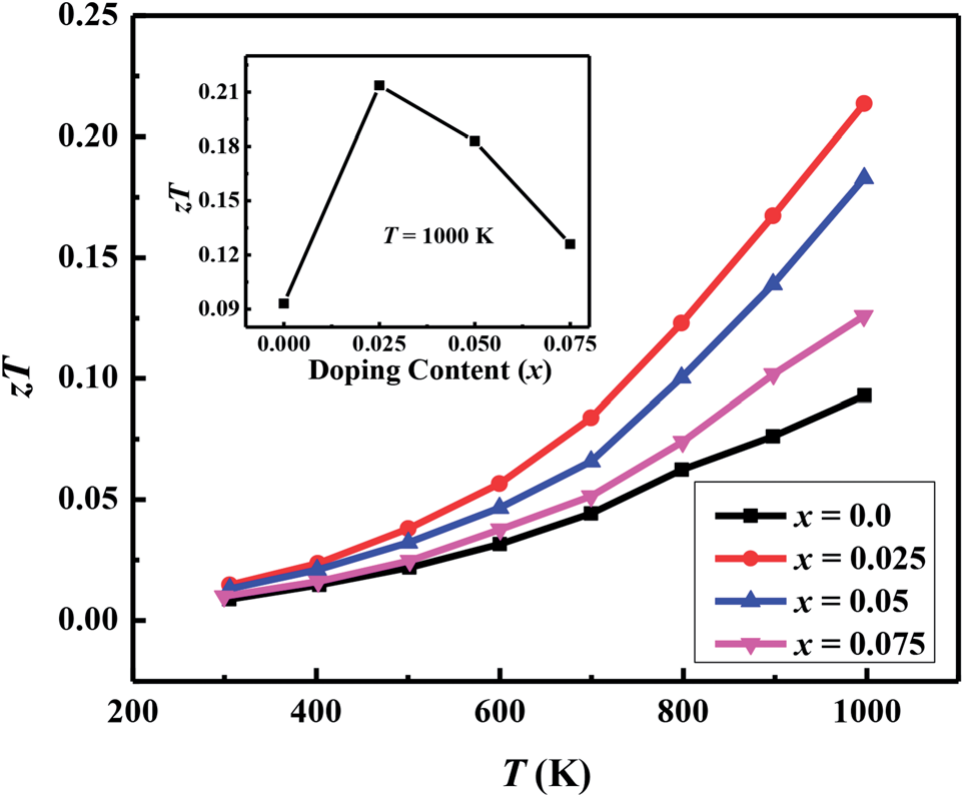
Thermoelectric figure of merit (*zT*) for Ca_3−2*x*_Na_2*x*_Co_4−*x*_W_*x*_O_9_ (0 ≤ *x* ≤ 0.075) samples as a function of temperature; inset shows doping content (*x*) dependence of *zT* at 1000 K.

The thermoelectric figure of merit (*zT* = *S*^2^/*κρ*) for Ca_3−2*x*_Na_2*x*_Co_4−*x*_W_*x*_O_9_ samples as a function of temperature and doping content (*x*) are shown in Fig. 10. It is evident that *zT* values of doped samples are significantly higher than the pristine C-349 system. The *x* = 0.025 has the highest *zT* value of 0.21 at 1000 K among all samples, which is about 2.3 times higher than *zT* value of the undoped sample. This increase in *zT* value is due to increase in Seebeck coefficient, and decrease in electrical resistivity and thermal conductivity of this sample. *zT* values of other doped samples are also reasonably good as listed in Table 2. As a comparison, *zT* value of our *x* = 0.025 sample is comparable or slightly better than previously reported results of Na doped Ca_2.5_Na_0.5_Co_4_O_9_ (*zT*_1000 K_ = 0.18),^25^ Bi and Na dual doped C-349 system (*zT*_1073 K_ = 0.18),^62^ Ca_2.8_La_0.2_Co_3.8_Cu_0.2_O_9_ (*zT*_773 K_ = 0.203),^40^ Bi and Na substituted Ca_3_Co_4_O_9_ system (*zT*_1000 K_ = 0.32),^26^ Gd and Y dual doped Ca_2.7_Gd_0.15_Y_0.15_Co_4_O_9+*δ*_ (*zT*_973 K_ = 0.26),^63^ NaF doped Ca_3−*x*_Na_*x*_Co_4_O_9−*x*_F_*x*_ (*zT*_873 K_ = 0.13)^64^ and Ca_2.9_Y_0.1_Co_3.97_Fe_0.03_O_9_ (*zT*_1000 K_ = 0.22) compound.^42^ It has been observed that electrical resistivity of our samples is still higher than most of the previously reported results, and therefore *zT* values of these materials are moderate. We believe that *zT* values of these samples can be further improved by preparing more compact materials under optimized synthesis conditions.

## 4. Conclusion

Polycrystalline samples of Ca_3−2*x*_Na_2*x*_Co_4−*x*_W_*x*_O_9_ (0 ≤ *x* ≤ 0.075) have been synthesized by the conventional solid state reaction method. Powder X-ray diffraction data revealed that Na^+^ and W^6+^ ions enter into the Ca_2_CoO_3_ and CoO_2_ layers of Ca_3_Co_4_O_9_ system respectively. High resolution XPS data showed that the average valence state of Co in our samples is between 3+ and 4+. Significant improvements in *ρ*, *S* and *κ* values of Na and W dual doped samples have been observed. These results are also supported by the carrier concentrations (*n*) and carrier mobility (*μ*) as confirmed by the Hall effect measurements. The observed power factor (PF) and thermoelectric figure of merit (*zT*) of 2.71 × 10^−4^ W m^−1^ K^−2^ and 0.21 respectively at 1000 K for *x* = 0.025 sample are comparable or slightly higher than most of the reported results of C-349 based materials, prepared by the conventional solid state reaction method. These results show that Na and W dual doping is an effective route for improving thermoelectric properties of Ca_3_Co_4_O_9_ system.

## Conflicts of interest

There are no conflicts to declare.

## Supplementary Material
